# Statistical characterization of multiple-reaction monitoring mass spectrometry (MRM-MS) assays for quantitative proteomics

**DOI:** 10.1186/1471-2105-13-S16-S9

**Published:** 2012-11-05

**Authors:** D R Mani, Susan E Abbatiello, Steven A Carr

**Affiliations:** 1 The Broad Institute of MIT and Harvard, 7 Cambridge Center, Cambridge, MA 02142, USA

**Keywords:** Multiple reaction monitoring mass spectrometry (MRM-MS), stable isotope dilution (SID), quantification, interference detection, limits of detection and quantification, intra- and interlaboratory precision

## Abstract

Multiple reaction monitoring mass spectrometry (MRM-MS) with stable isotope dilution (SID) is increasingly becoming a widely accepted assay for the quantification of proteins and peptides. These assays have shown great promise in relatively high throughput verification of candidate biomarkers. While the use of MRM-MS assays is well established in the small molecule realm, their introduction and use in proteomics is relatively recent. As such, statistical and computational methods for the analysis of MRM-MS data from proteins and peptides are still being developed. Based on our extensive experience with analyzing a wide range of SID-MRM-MS data, we set forth a methodology for analysis that encompasses significant aspects ranging from data quality assessment, assay characterization including calibration curves, limits of detection (LOD) and quantification (LOQ), and measurement of intra- and interlaboratory precision. We draw upon publicly available seminal datasets to illustrate our methods and algorithms.

## Introduction

In the past decade, the scientific community has seen an uptick in the use of mass spectrometry (MS) for the quantification of proteins and peptides in complex biological matrices. However, the technique that is most frequently used in quantitative assays, selected reaction monitoring (SRM, plural form: multiple reaction monitoring, MRM) MS was first reported in 1979 during the introduction of the triple quadrupole (QqQ) mass spectrometer [1]. Initially used for the detection, identification and quantification of small molecules [2-8], the QqQ has become prolific in proteomics laboratories and a necessary tool for the quantification of peptides and proteins, especially for biomarker verification. Biomarker verification is a step in the proteomics pipeline in which candidate biomarkers that have been identified from unbiased discovery experiments are targeted by quantitative assays utilizing stable isotope-dilution and MRM-MS [9]. This manuscript will focus on the statistical characterization and evaluation of MRM-MS assays arising from quantitative biomarker verification studies.

The power of the QqQ mass spectrometer comes from the inherent selectivity of its staged mass selection and detection. In the majority of quantitative MS experiments, the QqQ operates in SRM mode (plural form: multiple reaction monitoring, MRM). In this mode, as samples are ionized by electrospray ionization [10] and enter the instrument, the first quadrupole (Q1) is set to only allow the predefined m/z value of the precursor ion to pass into the second quadrupole, or the collision cell. In the collision cell, the selected ions enter a higher pressure region with argon or nitrogen gas, resulting in low energy collisions and fragmentation of the selected precursor ion into many product ions. Finally, only the preselected product ions with specific m/z values are allowed to pass through the third quadrupole (Q3) and on to the detector [1]. The result is a very selective means for separating the target ions away from everything that is being introduced into the mass spectrometer (i.e., through liquid chromatography or other sample introduction), and further detecting fragment ions of the target and reducing chemical noise from the sample. One of the benefits of MRM-MS on a QqQ MS platform is the speed at which it is able to detect multiple transitions (Q1/Q3 pairs), which is on the order of 10 msec per transition or less, allowing high multiplexing capabilities. This ability can be harnessed for both the analysis of many peptides (10's-100's) per assay, and the monitoring of many transitions per peptide. This ability is important because the identity of the peptide is reliant on the sparse few transitions that are detected and that discriminate it from other peptides or molecules in the sample. Therefore, a highly selective assay for a particular peptide would target several transitions, minimally three product ions. This results in three or more independent measures for a particular peptide target, which can make statistical analysis more complicated.

Due to the inherent instability of electrospray ionization, accurate and precise quantification is best achieved through the addition of a stable isotope-labeled standard (SIS) into the sample, an approach called isotope dilution [11-19]. The most common internal standards have been 13C and/or 15N-labeled peptide analogs, which introduce little chromatographic shift in reversed-phase chromatography so that they coelute with the target peptide and are chemically identical to the target peptide, except for the mass difference. The isotopically labeled standard is spiked into the sample as far up-stream in the sample handling process as possible. If isotopically labeled proteins are unavailable (such as uniformly 15N-incorporated proteins, or proteins with 13C and/or 15N modified amino acids such as arginine or lysine), then peptide analogs can be synthesized with isotopically labeled amino acids and spiked in pre- or post enzymatic digestion of the sample. These peptide standards behave similarly to the target peptide with regards to chromatographic separation, ionization, and fragmentation. The intensity of the signals detected for the SIS peptide is then compared to the signals for the analyte peptide, and their peak areas (determined from the area under the curve of the extracted ion chromatogram, XIC, for each transition) are compared to generate a peak area ratio (PAR). When the SIS peptide is spiked into the sample in a known quantity, the PAR is multiplied by the SIS peptide amount and the analyte peptide concentration is determined. While using only 3 transitions for the detection and identification of a target analyte seems sparse, the chromatographic retention times of the analyte peptide and SIS are also paired to ensure the proper peptide is detected. Finally, another important criterion to ensure peptide identity is the fragment ion ratio for a given peptide. This concept was first described in the context of small molecules as the "branching ratio", where each time a small molecule was fragmented and multiple product ions were detected, the ratio of these ions to one another was consistent: the largest fragment was always the largest, the smallest was always the smallest, and so on, as long as no interferences were present and the concentration was within the linear range of detection [6]. In the context of peptides, this effect is also seen from the fragmentation along the peptide backbone, and ensuring this ratio is consistent between the peptide target and the IS provides another level of selectivity and can indicate the presence of interfering signal [20]. This topic is further discussed below in Section 5.

While quantitative MRM-MS assays have been in practice for decades 3[4-7,11,15-19,21,23], this manuscript will focus on some more recent publications that use SID-MRM-MS for the quantification of peptides in plasma or similar complex matrices [15,22,23]. The first few examples describe the use of SID-MRM-MS for the quantification of peptides from samples with complex biological matrices [16-18]. In all cases, the work describes the use of SIS peptides as internal standards added to the sample matrix, sample analysis by MRM-MS and the calculation of peptide amount present in the sample. These papers created a turning point in the use of SID-MRM-MS in proteomics labs because they demonstrated the feasibility of simple assay development, throughput and precision in the quantification of target peptides present in complex samples.

The earlier publications on peptide quantification using SID-MS did not, in fact, have detailed sections on the statistical analysis of the quantitative data. Barr et al [15] report variances between MS run to MS run, or between digestion replicates, but did not discuss assay characteristics such as linear range or limits of detection and quantification. Gerber et al [16] briefly described the linearity of the assay between the concentration points assessed, but did not discuss reproducibility, the slope of the response curve or other metrics. Barnidge et al [17] showed the effect of equal weighting versus 1/x weighting when plotting the linear regression of the standard curve area versus concentration. Barnidge and Barr also discussed percent recovery of the peptide target from the proteolytic digest and sample handling, a topic that is further explored by Agger et al, for the quantification of apolipoproteins A-1 and B [24]. The more recent publications have more detailed sections on these calculations [15,19,20,23,24], but may still not be exhaustive enough to describe all aspects of calculations required to define an analytical assay for the newcomer. Therefore, this manuscript will consolidate many of the statistical and analytical approaches used to describe the quantitative aspects of SID-MRM-MS assays.

One example from a recent study--published in Nature Biotechnology, and hence referred to as the NBT study--evaluated the repeatability and reproducibility of SID-MRM-MS across multiple labs for the quantification of 10 peptides from 7 proteins spiked into human plasma [23]. The overall NBT study was constituted by three (sub-) studies. Study I is the peptide-level spike, where synthetic peptides were spiked into a background sample matrix of digested plasma to generate a calibration curve between 1 fmol and 500 fmol per μL in 1 μg of digested plasma. Study II is the protein-level spike, in which an equimolar mixture of the 7 target proteins were digested together, and then spiked into the background of digested plasma. This phase was designed to determine the effect of protein digestion on peptide recovery and its contribution to assay variability. The third phase, Study III, was also a protein-level spike, but mimicked a "real world" biomarker assay in which an equimolar protein solution was spiked into neat plasma and all subsequent sample handling steps (denaturation, reduction, alkylation, digestion, desalt and addition of stable isotope labeled peptide standards) were conducted at the individual laboratories. In all three cases, target peptides (or proteins) were spiked in at 9 different concentrations (1 fmol-500 fmol/μL in 1 μg/μL plasma) to generate a response curve, and the SIS peptides were spiked in at a fixed concentration of 50 fmol/μL in all samples including the blank, which consisted of digested plasma only. Eight laboratories participated in this study, seven of which used the same MRM-MS platform (4000 QTRAP, ABSciex) and the remaining lab used a TSQ Quantum Ultra QqQ (Thermofisher). All labs used nanoflow chromatography and adhered to an SOP that was distributed to dictate sample handling and data acquisition. All data acquired from this study is available on-line (http://www.proteomecommons.org/tranche/, Tranche hash: bCKpfN0bl2ULLwCaIovXn/spuw4rYfJF6H/L+/6sHAKGzCsj4fzTD0RauJjAwf9baB8tI36HQ0izji2tupYAPM29P2cAAAAAAAT0iw==), and will be used in example calculations.

Additional studies have been reported that aim to target clinically relevant analyte concentrations of proteins in plasma [15,22,24]. Of importance in these assays is measurement precision, inter- and intra-assay reproducibility or coefficient of variation (CV), as well as accuracy, and limits of detection and quantification. The following sections will discuss calculations of these parameters and metrics and discuss the necessary experimental design, as well as several methods for calculation and statistical analysis of the data. Many of these algorithms and calculations will be illustrated using the NBT study.

## Concepts and terminology for MRM-MS assay characterization

MRM-MS assays are characterized and evaluated based on several performance metrics and characteristics. Definitions of these metrics and associated terminology are laid out in this section, and will be used in the rest of the manuscript.

### Data

Peak areas from each of the monitored transitions (usually 3 or more per peptide form) are determined based on the extracted ion chromatograms (measured ion intensity or count per chromatographic time).

### Peak area ratio

In the context of SID-MRM-MS, the peak area of each peptide analyte transition is divided by the peak area of the corresponding transition from the stable isotope labeled peptide form to obtain the peak area ratio.

### Calibration curve

**Figure 1:**
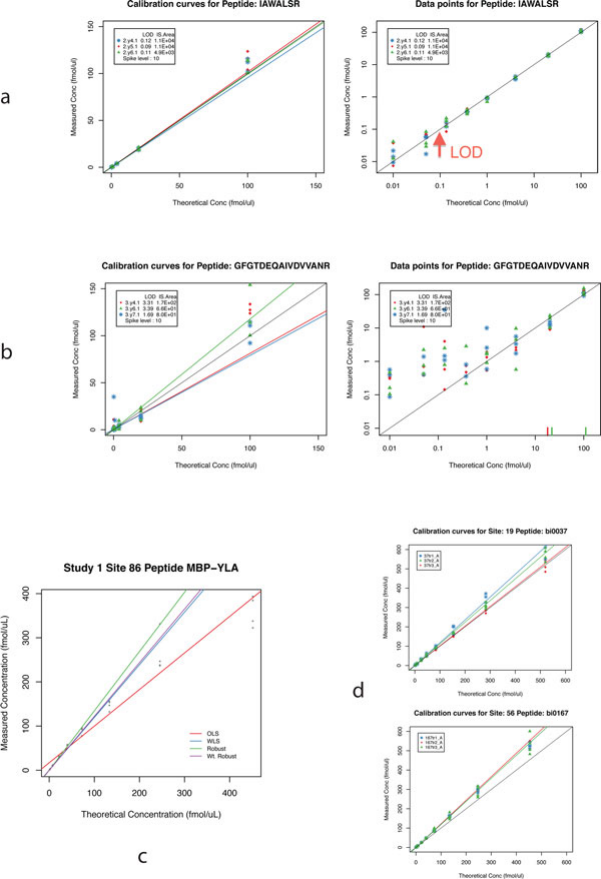
**(a) A set of calibration curves for 3 transitions of a well-behaved peptide, with a relatively low LOD and a linear response region spanning three orders of magnitude (n = 4 for each transition at all concentration points)**. The left panel shows the data points on the linear scale along with the calibration curves. The panel on the right shows the data on a logarithmic scale so that all points are clearly visible, along with the calculated LOD. **(b) **A set of calibration curves for 3 transitions of a poorly behaved peptide with significantly inconsistent measurements, resulting in a high LOD, and a very restricted linear response region. **(c) **Regression lines fitted using ordinary least squares (OLS), weighted least squares (WLS) where each point is weighted by the inverse square of its theoretical concentration, robust regression (using the MM-estimator) and weighted robust regression (MM-estimator with inverse square weighting). Weighted regression lines for least squares regression and robust regression are almost identical, with the robust regression line coming close. OLS is most affected by a few outliers. **(d) **Example calibration curves (i) site 19 transition 37tr1_A in blue on the top, and (ii) site 56 transition 167tr3_A in green (bottom), that have ideal slopes (i.e., slope = 1, see Table 1 and Section 3) when the regression line is fit using log-transformed data, but clearly have slope > 1 in linear space. The black diagonal line represents slope = 1 in the panels above.

Generally, a calibration curve is represented by the analytical response versus the concentration of a given analyte. For SID-MRM-MS experiments, a series of samples are analyzed that contain the sample matrix, a fixed concentration of SIS peptide, and varied concentration of the analyte peptide. The data are often plotted as "determined concentration" (or "measured concentration") versus "theoretical concentration" (see Figure 1), which will be used in the following examples. If the spike level of the internal standard is unknown, the data can also be plotted as peak area ratio versus theoretical concentration of the analyte. From the calibration curve, the slope is representative of the analytical sensitivity of the method for the analyte. Calibration curves are usually constructed so that the concentration spans at least two orders of magnitude and bracket the limit of detection and the upper limit of quantification.

### Precision

The precision of the data is determined by measuring replicates (3 or more) of one sample in the same manner. Precision is usually represented by standard deviation and coefficient of variation (CV).

### Accuracy

Accuracy of the data is calculated (when the true concentration is known) as percent error.

### Reproducibility

Synonymous with precision.

### Limit of Detection

The lowest analyte concentration at which the signal is discernable from the noise (chemical noise, white noise, etc), or detected with confidence [25]. This can be calculated in a variety of ways, several of which will be described below.

### Limit of Quantification

The lower limit of quantification refers to the lowest concentration of the analyte at which quantitative measurements can be made. The upper limit of quantification describes the highest concentration of analyte above which the signal departs from linearity. These two limits of quantification define the linear range of the assay.

### Overview

MRM-MS assays are used when the detection and quantification of specific analyte targets are required from a complex mixture. Stable isotope-labeled standard (SIS) peptides are used for a variety of reasons, but primarily act as an internal standard for the measurement of the peptide analyte and minimize the contributions of measurement variations due to chromatography, ionization, fragmentation and detection by MS. Assays can be designed to determine the Figures of Merit (limits of detection and quantification, precision and accuracy) by incorporating a calibration curve. The Figures of Merit can change due to differences in sample matrix (both nature of matrix and concentration) and factors affecting instrument sensitivity (chromatographic resolution, ionization, MS detection, etc). It is recommended to determine Figures of Merit if any of these factors are changed, and periodically on the same instrument, especially when analyzing samples that will be detected near the lower LOQ of the assay or when high precision is required.

In typical quantitative SID-MRM-MS assays, the determined Figures of Merit are strongly influenced by system performance, both in terms of sensitivity and reproducibility from sample to sample. The noise contributed by the sample matrix also plays a major role in the magnitude of the calculated LOD and LOQ, and this is determined usually by several (at least three, preferably more) repeat measurements of matrix blanks (sample including everything except the target analyte) run throughout the course of the assay. With current technologies and on normally functioning nanoflow LC-MRM-MS systems, typical peptide LODs can be attained in the 100's amol per 1 ug equivalent protein digest load [19,23].

## Calibration curve and regression analysis

The starting point of most quantitative assays is the calibration curve (Figure 1a and 1b). A range of analyte concentrations is analyzed in sample matrix to define the linear range of detection, limits of detection and quantification, and reproducibility of the assay. The calibration curve is designed to explore the possibility of endogenous signal in the matrix by multiple measurements of a blank sample (sample matrix and internal standard), and to also determine if there is interference in the analyte signal. In the case of SID-MRM-MS, the SIS peptide is always present in the sample to normalize for any instrument-related issues that may affect analyte detection. It may be spiked in upstream in the workflow to also account for losses due to sample handling. The SIS peptide is spiked in at a known concentration to determine the concentration of the target analyte. With the target analyte spiked in at specific concentration and the SIS peptide spiked in at a fixed, known amount, the peak area ratio (PAR)--the ratio of peak intensities of the analyte to standard--is proportional to the concentration of the analyte. The measured concentration is then calculated as the product of the PAR and the concentration of the heavy standard:

measured concentration=PAR×concentration of heavy standard

When the target analyte is spiked in at various concentrations spanning a range of values, we obtain a set of measured concentrations corresponding to the spiked-in theoretical concentration. A linear calibration curve relating the theoretical and measured concentration can be fitted:

measured concentration=slope×theoretical concentration+y-intercept

An ideal calibration curve has a slope of 1 and an intercept of 0, indicating that the measured concentrations are in excellent agreement with the theoretical concentrations. An example of a well-behaved peptide is shown in Figure 1a. Deviation of the slope from 1 indicates less than ideal response, and a significant non-zero intercept is indicative of the presence of endogenous analyte (Section 4.1 and 4.2).

A standard way to fit such calibration curves is ordinary least squares (OLS) regression [11]. While non-linear calibration curves could also be fitted, such curves may tend to overfit the data, given the relatively small number of points used for the fit. Furthermore, the slope and y-intercept of a linear regression fit have additional relevance from a quantification perspective.

MRM-MS assays usually have a linear operating region where the intensity response linearly varies as the spike-in concentration of the target analyte is varied. When a concentration curve is run, these limits of the linear region are not known--in fact determining this region is one of the goals of running the response curve. As such, we expect some analyte concentration values at the high and/or low end of the spectrum to lie outside the linear operating region. Therefore, when a linear OLS regression curve is fit, these points in non-linear regions of the MRM-MS response can unduly affect the regression fitting, resulting in skewed slope and y-intercept values. Robust regression [2,14] is one approach used to address this problem. Robust regression fitting algorithms are resistant to outliers, and down-weight points that deviate from the main bulk of data points, resulting in more reliable estimates. Some common methods for robust regression includes least median of squares (LMS) regression, least trimmed squares (LTS) regression [26] and the use of the MM-estimator [27].

Furthermore, the variance of concentration measurements tends to increase at higher concentrations. In order to account for this trend data points are weighted according to the inverse square of the measurement or variance at that measurement level. This weighting can be used either with least squares regression (resulting in weighted least squares, or WLS regression) or with robust regression.

A comparison of OLS, WLS and robust regression with and without weighting for representative peptides in the NBT Study data are shown in Figure 1c. As is evident from the Figure, OLS is significantly influenced by the few points at the higher concentration level. Robust regression is more resistant to such outlier and tends to fit the regression line to follow the trend captured by a majority of points. The weighted regression lines for WLS and robust regression are much closer and are significantly less influenced by outliers and the high variance at the upper end of the calibration curve.

An alternative to WLS or weighted robust regression is to fit the regression line on log-transformed data [36], where the logarithms of both the measured and theoretical concentrations are used as the data points for the regression. The log transform converts heteroscedastic data into a homoscedastic set [11] thereby eliminating the dependence of variance on the concentration values. But regression lines fit in log space tend to downplay the deviation of the observed data from ideal, resulting in slopes that are closer to 1, and hence could provide an incorrect impression that the assay performance is better than it actually is (see Table 1, and Figure 1d). In addition, with log transformation, the intuitive interpretation of regression slope as sensitivity (see below) and intercept as endogenous level (see Section 4.1 and 4.2) are no longer valid, making it harder to interpret the results. Plotting the data on a log-log scale, on the other hand, enables more effective visualization since such a plot can naturally accommodate the concentration variation range normally used in such experiments (although the non-log-transformed linear regression line cannot be conveniently plotted, and may appear as a non-linear curve).

**Table 1:** Comparison of the regression analysis in linear and log space.

	Regression slope after log-transformation	
Regression slope in linear space	not ideal	ideal (slope = 1)
not ideal	167	53
ideal (slope = 1)	14	6

Measured and theoretical concentrations from NBT Study I (with 8 sites, 10 peptides and 3 transitions per peptide) are natural log transformed (other common bases used for the log transform are 2 and 10). Weighted, robust regression lines are fitted to the linear data while robust regression is used for log-transformed data. The slope of each regression is assumed to be 1 (ideal) if the 95% confidence interval calculated as slope ± t_(1-Δ/2),df _* *s.e. *includes 1 (where, t_(1-Δ/2),df _ is the 2-tailed t-distribution critical value for α = 0.05, df = (# replicates - 1), and *s.e. *is the standard error for the regression slope). As is evident from the table, log-transforming data results in more ideal slopes (closer to 1). Visual inspection of the regression lines and associated data indicate that this overestimation of the number of ideal peptides by log-transformed regression is unwarranted and misleading, as illustrated by examples in Figure 1d.

Traditionally in analytical chemistry, the slope of a linear regression is related to the sensitivity of an assay, which describes the ability of the assay to differentiate between small changes in analyte concentration [23]. Calibration sensitivity is equal to the slope of the calibration curve and is independent of concentration. This definition is the quantitative definition of sensitivity that is recognized by the International Union of Pure and Applied Chemists (IUPAC). Calibration sensitivity, however, does not take into account measurement precision. Analytical sensitivity (γ), described by Mandel and Stiehler [29], takes into account the precision of the measurements as well as the slope of the calibration curve: γ = *m*/s_s_, where *m *is the slope of the calibration curve and s_s _is the standard deviation of the measurement. In the context of peptide quantification, the slope of the calibration curve or the analytical sensitivity would easily aid in the selection of the best peptide targets, if there were several to choose from, and is also a good measure of whether or not similar instruments are measuring the target peptides with equal sensitivity. However, in addition to sensitivity, other figures of merit can be calculated from these values, including limit of detection, limit of quantification, and the amount of endogenous signal present in the blank [23].

Given the importance of the slope and intercept of the regression line for the calibration curve, an additional approach to evaluating the robustness and quality of the regression fit is to inspect the 95% confidence intervals for the slope and intercept. While many regression fitting algorithms provide an estimate of the standard error, the 95% confidence intervals can be easily calculated [27]. Bootstrap resampling is an alternative method for determining these limits [14] (also see Section 4.2 below).

Currently, less attention has been given to slope and y-intercept, and are often not reported in publications, in lieu of R^2 ^[30]. R^2 ^is a measure of "explained variance", and does not provide an indication of the robustness of the regression fit. In addition to R^2^, other factors of the regression fit including confidence intervals of the slope and intercept, residuals and a graph of the data should be examined before judging the quality of the regression line [31].

## Limits of detection and quantification

Limits of detection (LOD) and quantification (LOQ) are important characteristics of any quantitative method, and in the MRM-MS assay can be determined using the calibration curve. The intuition and definitions related to LOD and LOQ determination are described in Currie, 1968 [31]. There are a variety of methods to calculate LOD and LOQ, based on different aspects of the assay, and its intended application. A brief summary of the different classes of methods to determine detection and quantification limits is given below.

### Blank Sample

In this approach, replicates of a blank sample--i.e., a sample with the target analyte absent--are used to determine the LOD and LOQ of the analyte [31]. Assuming that random measurement errors are normally distributed, and with 5% risk of incorrectly claiming detection in the absence of analyte (α) or missing the detection of analyte (β), LOD = 3.29 σ_B _and LOQ = 3 × LOD = 10 σ_B _where σ_B _is the standard deviation of the blank sample.

### Blank and Low Concentration Sample

The above method uses only the blank sample. In practice, the standard deviation of the blank sample could be significantly different from the standard deviation with the analyte present at a low level. To account for this possibility, LOD and LOQ calculation explicitly takes both the blank and the low concentration samples into account. A variation of the partly nonparametric method in [10] is to use a parametric approximation to account for a small number of replicates. This approach used in [23] is to calculate LOD as: LOD = μ_B _+t_(1-β) _(σ_B _+ σ_S_)/√n, where μ_B _is the estimated mean of the blank samples, σ_B _is the standard deviation of the blank samples and σ_S _is the standard deviation of the low concentration samples. The equation assumes that analyte concentration is estimated using the mean of n replicates. Given the LOD, LOQ is estimated as 3 × LOD.

### Calibration Curve

Instead of using just the blank or a low concentration point, this method uses the entire calibration curve to determine LOD. Also termed the calibration plot method, the standard error s_y|x _of the measured concentration (y-estimate in the regression equation) is used in place of the standard deviation of the blank sample [32]. The LOD is then calculated as LOD = 3 s_y|x_/slope, and LOQ = 3 LOD.

### RSD Limit

This approach [33] determines LOQ based on an accepted target value for relative standard deviation (RSD). RSD is the absolute value of the coefficient of variation (CV, the ratio of standard deviation to mean), and is expected to be small at the LOQ (typically less than 10% or 20%). The calibration curve is used to determine the RSD at each spike-in level, and the RSD variation is modeled as a function of the analyte concentration using RSD = *level *× p_1_^(1 - p^_2_^1og(*level*))^. The parameters p_1 _and p_2 _are determined using a fitting process, and the LOQ is that analyte concentration where the target RSD is achieved. The LOD is then reported as LOD = LOQ/3.

**Figure 2:**
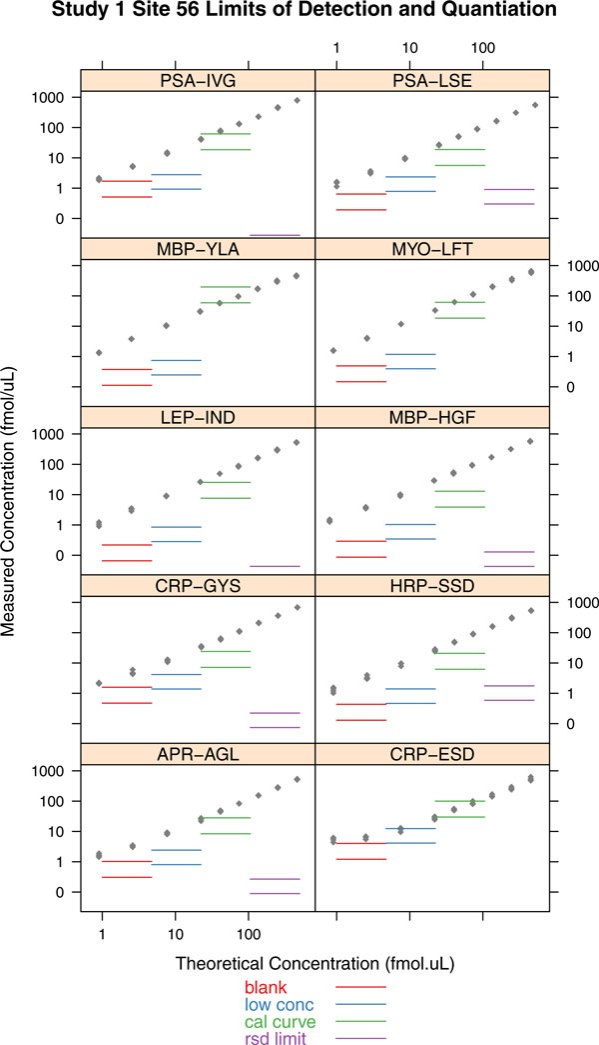
**A comparison of the various methods for calculating limit of detection (LOD, lower line in a pair) and limit of quantification (LOQ, upper line in a pair)**. The four methods compared are described in Section 4. The method using blank + low concentration sample is the most reliable, and consistently produces acceptable LOD and LOQ values for most practical purposes. The blank only method is a close second, but can under-estimate the LOD and LOQ. The calibration curve method results in very conservative estimates, while the RSD limit method is inconsistent with some extremely low LOD and LOQ values.

Figure 2 shows a comparison of the four methods for determining LOD and LOQ for representative peptides from the NBT Study. The calibration curve method generally tends to overestimate the LOD. The RSD Limit method tends to significantly underestimate LOD, resulting in extremely low LOD and LOQ value for many peptides (e.g, MYO-LFT, CRP_ESD, LEP-IND). The blank sample approach and the blank+low concentration sample method result in approximately similar values, with the blank sample method usually resulting in lower limits. Based on this evaluation, we have chosen the blank and low concentration sample algorithm as the preferred method for determining LOD and LOQ. This method is simple to implement and does not require the generation of the entire calibration curve.

Endogenous presence of analyte signal in the sample matrix is a difficult problem to deal with because it can complicate the calculation of LOD and LOQ. In addition, any signal derived from a spiked-in analyte (as in a calibration curve experiment) is added to the endogenous signal. One experimental approach to circumvent this issue is to use a surrogate matrix, one that is very similar to the sample matrix, but does not contain the endogenous analyte. This can be difficult to find, especially in a sample matrix as complex as plasma with thousands of proteins ranging ten orders of magnitude of concentration [34]. Using plasma from a different species may even introduce new problems, such as interfering signal. An experimental alternative is to use the internal standard as a surrogate analyte and vary its concentration in the sample matrix to generate a standard curve [35]. While a reasonable alternative, this can cause questions to arise about the difference in the chemical noise that may be present at the m/z values for the surrogate analyte versus the real analyte. A stable isotope-labeled version of a peptide with a mass shift of 6 amu may have an entirely different level of chemical noise contributed by the sample matrix and electronic noise. Therefore, it is beneficial to consider a statistical means of estimating the endogenous level of analyte present in a sample matrix.

Endogenous levels of an analyte present in the LC-MS matrix can be estimated using the linear range of the calibration curve resulting from a dilution or standard addition experiment [15]. Using the input data consisting of measured concentration values for corresponding calibration curve (theoretical, or true) concentrations, a robust linear fit using least median squares regression [14] is performed to determine the regression line y-intercept for:

measured concentration=slope×theoretical concentration+y-intercept

with the y-axis representing measured concentration and the x-axis representing theoretical concentration. The 99% confidence interval of the regression line y-intercept is calculated using bootstrap estimation with repeated (1000 or more) resampling iterations [28]. The bootstrap estimation involves resampling with replacement from the data, in order to assess expected variation. For each resampled data set, the regression above is re-fit to recalculate the slope. The basic non-parametric confidence interval [28] for the slope is estimated as the range (*m*_α_, *m*_1-α_), where *m_p _*is the *p*-th percentile of the slope in the resampled estimation, and (1-2α) is the confidence level. Usually, α = 0.025 or 0.005, for a confidence level of 95% or 99% respectively.

If the lower limit of the confidence interval is positive, then the analyte is deemed to have an endogenous level equal to the regression y-intercept. If the lower 99% confidence interval is zero or negative, there is no expected endogenous level for that analyte. Once endogenous levels (if present) are calculated, the estimated LOD (and hence LOQ) in the absence of endogenous analyte is the difference of the calculated LOD (in the matrix) and the estimated endogenous level.

The method has been applied to selected transitions (the "best" transition that provides the lowest LOD) of the peptides for which MRM assays have been configured in [15]. Of the 28 peptide transitions analyzed, 3 are reported to have endogenous levels (see Table 2). The CRP peptides (bi0090 and bi0092) are expected to have endogenous levels. Thus, the only false positive is peptide bi0119 for MRP14, with an estimated endogenous level of 0.28 fmol. When all the 84 transitions monitored for these 28 peptides are considered, 16 transitions have reported endogenous levels. Of the 16, six of the transitions are for CRP peptides where an endogenous level is expected. Another 5 transitions have some form of interference (as is evident from the unusually large LOD/LOQ values of these transitions compared to other transitions for the respective peptide), and the interference is interpreted as an endogenous signal--these are situations that can be avoided by using AuDIT [33] (see Section 5). The remaining 5 transitions listed as having endogenous levels appear to be false positives.

**Table 2:** Summary of endogenous calculations for 28 peptides from 8 proteins.

Protein	Peptide	Sequence	LOD	LOQ	Endogenous Level
Natriuretic peptides B	bi0096	MVLYTLR	0.082	0.246	0
	bi0097	M_(ox)_VLYTLR	0.098	0.294	0
	bi0098	ISSSSGLGCK	0.074	0.223	0
	bi0099	MVQGSGCFGR	0.081	0.244	0
	bi0100	M_(ox)_VQGSGCFGR	0.216	0.647	0

CRP	bi0090	ESDTSYVSLK	1.454	4.361	1.072
	bi0092	GYSIFSYATK	1.949	5.847	1.838

IL33	bi0120	DNHLALIK	0.606	1.818	0
	bi0121	TDPGVFIGVK	0.172	0.515	0
	bi0122	DFWLHANNK	0.271	0.812	0
	bi0123	VLLSYYESQHPSNESGDGVDGK	0.274	0.822	0

MCP1	bi0124	WVQDSMDHLDK	4.523	13.568	0

MPO	bi0102	IPCFLAGDTR	0.287	0.862	0
	bi0104	IANVFTNAFR	0.329	0.988	0

MRP14	bi0118	LTWASHEK	0.397	1.190	0
	bi0119	LGHPDTLNQGEFK	0.475	1.425	0.283

sCD40L	bi0108	SQFEGFVK	0.203	0.609	0
	bi0105	TTSVLQWAEK	0.202	0.606	0
	bi0106	EASSQAPFIASLCLK	0.086	0.257	0
	bi0109	SLSLLNCEEIK	0.071	0.212	0

Troponin	bi0082	TLLLQIAK	0.088	0.265	0
	bi0083	NITEIADLTQK	0.055	0.165	0
	bi0084	NIDALSGMEGR	0.256	0.767	0
	bi0086	VLAIDHLNEDQLR	0.154	0.461	0
	bi0087	SFMPNLVPPK	0.108	0.324	0
	bi0088	SFM_(ox)_PNLVPPK	0.116	0.349	0
	bi0089	YEINVLR	0.052	0.157	0

See section 4.2 for description.

There have been no observed instances of false negatives where an endogenous level was expected, and the method returned with a 0 endogenous level. If such instances are encountered, the confidence interval can be relaxed to 95% (from the currently used 99%) to enable overcoming false negatives (at the expense of more false positives).

Effective application of the method is dependent on having enough points on the concentration curve that are in the linear operating range. If there are too few points in the concentration curve, or if the endogenous level is so high that most of the concentration curve is non-linear and affected by endogenous analyte, the method will fail. Theoretically, the method is likely to succeed if at least 50% of points on the concentration curve fall in the linear operating range (since least median squares regression has a breakdown point of 0.5).

## Imprecision and interference in MRM MS

**Figure 3:**
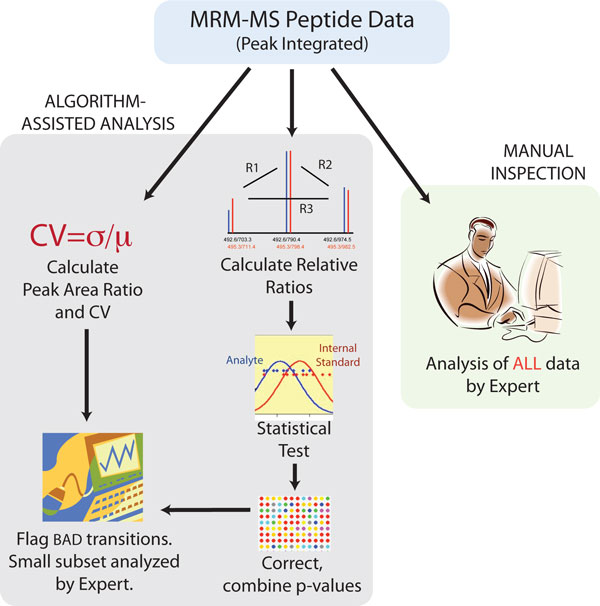
**Analysis work flow for isotope dilution MRM-MS data with and without the use of AuDIT**. After LC-MRM-MS analysis of samples, transition peaks are identified and integrated with software from either the mass spectrometer vendor or another supplier. (A), Flow of data with use of the automated algorithm. The statistical test identifies problem transitions from the variation in the relative ratios for the analyte and the SIS. The CV of the PARs is used as a filter to flag transitions with unacceptably large variation. (B), The current standard practice of careful manual inspection of all transitions by an expert. Adapted from Abbatiello, Mani, et. al., Clinical Chemistry, 56, 291-305 [17].

Inaccurate quantification in peptide MRM-MS can result from many factors including incorrect peptide identification, matrix suppression, interference in one or more of the product ion transitions monitored, poor chromatography, MS-instrument related signal attenuation and saturation, and errors introduced during peak detection and integration (Table 3). Interferences in MRM-MS from such sources are usually detected by painstaking and subjective manual examination of raw data [36]. Protein quantification for candidate biomarker verification in clinical proteomics [15,18,19,22,37] and other relatively high throughput applications increasingly require the ability to assay for many 10's to hundreds of proteins. Clearly, manual inspection of such data is no longer possible nor desired. The quality assessment of MRM-MS data can be automated using AuDIT, an algorithm for *Au*tomated *D*etection of *I*naccurate and imprecise *T*ransitions in MRM-MS for quantitative peptide analyses in any biological matrix, and can be used both in method development as well as for routine testing of patient samples [33]. AuDIT greatly increases the speed, reliability and accuracy of peptide identification and quantification from MRM-MS data analysis. Figure 3 shows the analysis workflow for using AuDIT.

**Table 3:** Summary of potential problems encountered during analysis of SID-MRM-MS data that often require manual identification or re-integration and their effect on the precision and accuracy of quantification.

Data Issue	Impact on Quantification
**Poor chromatographic peak shape**	Imprecise and inaccurate area assessment
**Chromatographic peak too narrow (<6 points across)**	Imprecise and inaccurate area assessment
**Detector saturation**	Inaccurate peak area assessment
**Inconsistent integration between analyte and SIS peptides**	Imprecise and inaccurate peak area assessment
**Interference in analyte or SIS signals**	Inaccurate peak area assessment

AuDIT was designed to extensively use the concept of "relative ratio" or "branching ratio" [6] defined as the ratio of the peak areas for any 2 transitions of the same precursor. All analyte (or all SIS) transition peak areas are used in pairs to calculate the ratio. The relative ratio is unlike the PAR, which is calculated as the ratio of analyte to SIS peak areas for a given transition of a specified precursor. The AuDIT algorithm operates on preprocessed data and executes the following steps:

1. Use all transitions of a peptide (peak area from XICs) to calculate relative ratios by either the minimal-pairs or all-pairs method. The minimal-pairs method calculates the relative ratio of a given transition by dividing its peak area by the peak area of one other transition from the same precursor. The all-pairs method calculates ratios for all possible transition pairs generated from one precursor. This process is performed for each peptide analyte and its corresponding SIS so that the relative ratios of the analyte can be compared with the relative ratios of the SIS.

2. Apply the t-test to determine a p-value for the hypothesis that the relative ratios for the analyte are different from the relative ratios of the SIS.

3. Use the Benjamini-Hochberg false-discovery rate method to correct the nominal t-test p-values to account for multiple hypothesis testing [38].

4. Disaggregate the corrected p-values for the relative ratios into combined p-values for each transition. Each transition is used to calculate either 2 ratios for the minimal-pairs method or n-1 ratios for the all-pairs method (where n is the total number of observed transitions for each peptide). Calculation of the p-value for determining if a transition is problematic requires combining the p-values for the respective relative ratios. Because the same peak areas from a given transition were used in calculating all its ratios, the resulting p-values are not independent. These dependent p-values are combined by means of a previously outlined methodology [19,39].

5. Calculate the CV for the PAR (analyte/SIS) from the results for all replicates in a transition for a given sample.

6. A transition is marked as "bad" if either the corrected combined p-value for the transition is less than the p-value threshold of 10^-5 ^or if the CV is greater than the CV threshold of 0.2 (20%). Transitions not satisfying either of these conditions are classified as "good." Although the chosen thresholds work well for many data sets, they can be changed to fine-tune the algorithm as needed.

There are currently no automated methods for identifying transitions with interferences (or other problems, see Table 3) that can render them unsuitable for quantification. As such, the final decision on the quality of a transition is subjective and has relied entirely on expert review of the data [26]. In order to evaluate our algorithm for inaccurate and imprecise transition detection, we compared the results of AuDIT with that of an expert using a two-step process. In the preliminary phase, the expert looks at all the integrated extracted ion chromatograms, and creates an unbiased "pre-algorithm" annotation which records any potential problems the expert observes like poor chromatography, inaccurate peak integration, etc., and is recorded at the level of the MRM transition. The data is then run through AuDIT, and the 'good' or 'bad' classification of the algorithm is compared with the expert's annotation ("global", in Table 4). In cases where AuDIT's decision and the expert's annotation disagree, the expert re-evaluates those transitions to see if AuDIT's assessment (good or bad) is justifiable--i.e., the actual observations of questionable data quality or interferences are such that the relative ratio may not be affected (and hence the transition may be used for quantification), or vice versa. This final phase of expert review creates a "post-algorithm" annotation, and is performed with the same criteria and rigor as the first review, but primed for issues that might have been overlooked or wrongly assessed. This focused annotation is compared with AuDIT decisions to evaluate its efficacy in identifying inaccurate transitions, and the overall performance is summarized in Table 4.

**Table 4:** Validation of AuDIT.

Dataset		Annotation	TN	TP	FN	FP	Overall Accuracy (%)	Sensitivity (%)	Specificity (%)
**10 Peptide Standard Curve, 3 transitions MultiQuant**	Site 1	Global	89 11	119	29	33	77	80	73
		Focused	7	144	1	8	97	99	94
	
	Site 2	Global	9	217	14	30	84	94	23
		Focused	23	247	0	0	100	100	100
	
	Site 3	Global	19	200	33	18	81	86	51
		Focused	50	218	2	0	99	99	100
	
	Site 4	Global	21	162	74	13	68	69	62
		Focused	81	174	14	1	94	93	99

**10 Peptide Standard Curve, 3 transitions, Skyline**	Site 1	Global	29	163	35	43	71	82	40
		Focused	56	206	8	0	97	96	100
	
	Site 2	Global	1	210	15	44	78	93	2
		Focused	15	254	1	0	100	100	100
	
	Site 5	Global	35	34	2	199	26	94	15
		Focused	37	232	0	1	100	100	97

**10 Peptide Standard Curve, 5 transitions, MultiQuant**	Site 6	Global	46 16	277	122	23	69	69	67
		Focused	8	294	0	6	99	100	97

**Clinical Samples, 3 transitions, MultiQuant**	Cardio-vascular Peptides	Global	4	33	5	9	73	87	31
		Focused	9	40	0	2	96	100	82

For each dataset, two contingency matrices are calculated. The 'pre-test' evaluation by the expert identifies overall data problems like poor chromatography, inaccurate peak integration, etc. Comparison of this **global **annotation with the algorithm calls results in one set of contingency matrices (shown under Annotation = Global). The second 'post-test' re-evaluation is based on the algorithm outcome, and accounts for the fact that the global annotation could be overly conservative (i.e., mark too many transitions as BAD). This **focused **annotation is compared with the algorithm-derived decisions to derive a second, algorithm-guided set of contingency matrices, shown under Annotation = Focused. TN: True Negative, TP: True Positive, FN: False Negative, and FP: False Positive. Overall Accuracy = (TP + TN)/(TP + TN + FN + FP). Sensitivity = TP/(TP + FN). Specificity = TN/(TN + FP). A transition is BAD if it has some form of interference, i.e., it is imprecise of inaccurate. If not, the transition is labeled as GOOD. Adapted from Abbatiello, Mani, et. al., Clinical Chemistry, 56, 291-305 [17].

**Figure 4:**
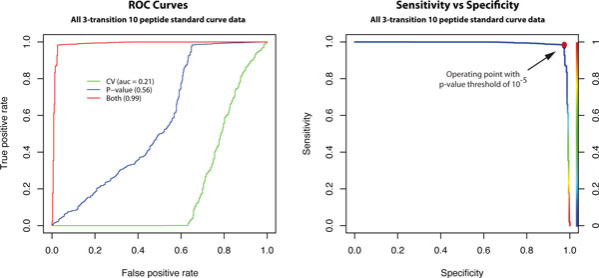
**ROC curve and sensitivity-specificity plots summarizing performance of AuDIT in identifying inaccurate and imprecise transitions, as evaluated by an expert**. AuDIT uses the t-test p-value and the CV of the PAR (ratio of analyte peak area to SIS peak area) to detect problem transitions. (A), Both the p-value and the CV are required to achieve acceptable performance (i.e., as indicated by AUC values in parentheses). (B), Specificity and sensitivity values achieved as the p-value threshold is varied from 0 to 1 (with a fixed CV threshold of 20%). The chosen p-value threshold of 10^-5 ^used for all of the analyzed data is indicated by the red circle (sensitivity, 98%; specificity, 97%). The rainbow color bar (right y axis) keys the location of the p-value threshold on the sensitivity-specificity curve. Adapted from Abbatiello, Mani, et. al., Clinical Chemistry, 56, 291-305 (22).

A 2 × 2 contingency matrix is created to evaluate the performance of AuDIT on each dataset. Defining a 'positive' as a 'good' transition call, the Table shows the various elements of the contingency matrix. Algorithm performance is estimated using i) overall accuracy, ii) sensitivity and iii) specificity, as described in Table 4. A receiver operating curve (ROC) is used to evaluate the combined effect of incorporating p-value and CV in the algorithm calculations for flagging inaccurate and imprecise peptides (Figure 4). The area under the ROC curve (AUC) is an indication of the quality of the classifier [40]. The ROC and AUC also show that the t-test and the CV jointly achieve significantly better performance than either measure alone. The AUC of the ROC curve for transition quality prediction using only the CV is less than 0.5, indicating that this modality is worse than a random predictor. The CV is affected only under specific circumstances, most of which are orthogonal to situations where a significant t-test score will be obtained. It is therefore imperative that both the t-test and the CV be used in order to derive an accurate predictor of imprecise or inaccurate transitions. The p-value for these experiments was set to 10^-5 ^and the CV value set to 0.2. Both the p-value and CV thresholds are adjustable. While the CV was set to an arbitrary value of 0.2, a sensitivity-specificity curve (Figure 4b) was used to assess the effect of changing the p-value threshold for comparison of the relative ratios of the fragment ions between the analyte and SIS peptides. As the p-value threshold increases above 10^-5^, a concomitant decrease in sensitivity is observed. At p-values lower than 10^-5^, the specificity of the algorithm decreases. Thus, a p-value of 10^-5 ^was selected as the optimum threshold for sensitivity and specificity of the algorithm for identification of inaccurate or imprecise transitions.

AuDIT can be applied to data exported from most MRM-MS analysis software, and can potentially be embedded into such applications to greatly reduce manual inspection and alert the researcher of potentially errant data at an early point in the data analysis, potentially allowing problematic samples that exhibited large CV values (for example, maybe caused by column degradation and poor peak shape) to be re-acquired. In addition, incorporation of AuDIT into the MRM-MS workflow would streamline the processing, likely resulting in more efficient generation of accurate and precise quantitative data from SID-MRM-MS analyses.

The AuDIT software is available at http://www.broadinstitute.org/cancer/software/genepattern/modules/AuDIT.html.

AuDIT provides a mechanism to evaluate SID-MRM-MS data quality from the perspective of minimizing interferences to enable robust quantification. A complementary approach involves assigning quality scores to the MRM-MS spectra in order to statistically define error rates for peptide identities, as implemented in mProphet [41]. mProphet uses characteristics of the transition peaks and the concept of "decoy peaks" (measured where no real peaks are present) to derive a composite discriminant score that statistically captures the quality and reliability of the MRM-MS data for each peptide.

In addition to AuDIT and mProphet, other data analysis software packages possess features that help to evaluate the composite signal of all transitions measured for a peptide and its SIS to monitor for differences. Such features are available in Skyline [21], a vendor neutral data analysis program, by monitoring the signal contribution from each transition and enabling the user to compare it to that of the SIS peptide with the output in visual plots. PinPoint software (Thermo Fisher Scientific) also compares the fragment ion ratio of the light and heavy peptides to look for agreement and reports the fragment ion ratios for the light and heavy peptides, also with visual plots. These software features work well for detection of interfering signal in a transition from a given peptide, and through the use of visual plots enable rapid screening of large data sets with a variety of peptide targets.

## Intra- and interlaboratory variation

In order for MRM-MS combined with stable isotope dilution to be used as an assay for quantitative measurement of proteins and peptides, the precision and variability of the assay needs to be characterized not only in a given laboratory, but also across multiple laboratories. Assessment of the intra- and inter-lab variation of MRM-MS assays was the primary goal of the NBT Study described in Section 2.

**Figure 5:**
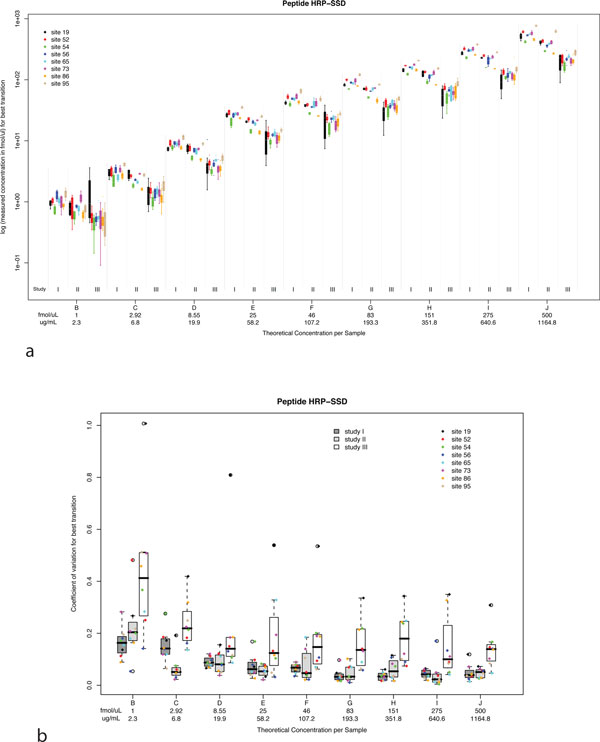
**Box plots of variation in MRM quantitative measurements, interlaboratory CV, and intralaboratory CV**. All CV calculations are performed on the original data, while log-scaled axes are used to enchance visualization in the plots. (a) Intralaboratory assay CV. Box plots showing measured log concentration (y axis) versus theoretical (spiked-in) concentration (x axis) for HRP-SSD across the entire concentration range in diluted plasma. Protein concentration in mg/ml is mg protein equivalent in 1 ml undiluted plasma. The box plots for studies I and II are based on four replicate measurements, whereas those for study III summarize 12 measurements (four from each of 3 process replicates). Each of the eight sites was assigned a random numerical code (19, 52, 54, 56, 65, 73, 86, 95) for anonymization. (b) Interlaboratory assay CV. Values are shown for studies I-III for the entire range of HRP-SSD final analyte concentrations in plasma. Within each box plot, actual intralaboratory CV values for individual laboratories are shown with color-coded markers. The CV values are calculated based on the single best performing transition (lowest combined CV) across studies I and II. This same transition is also used for study III. Adapted from Addona, et. al., Nature Biotechnology, 27(7):633-41 [23].

In the NBT study, intralaboratory variability and reproducibility in studies I-III were evaluated by comparing the measured concentrations to the theoretical concentrations across the range of spiked-in analytes and determining the coefficient of variation (CV = standard deviation/mean) for these quantitative measurements. In addition to calculating CV, graphical visualization [29] can assist in analysis and provide insight on variation across concentration levels, study phases and different laboratories. Figure 5a shows measured log concentration (y axis) versus theoretical (spiked-in) concentration (x axis) for the SSDLVALSGGHTFGK peptide derived from horseradish peroxidase (HRP-SSD). Data for each site are color- coded, and organized by study phase and concentration. As expected, a linear trend is observed in the measured concentrations for studies I-III as spiked-in analytes increase across the concentration range. However, measured concentrations decrease as laboratories progress from study I to II to III. This trend is a result of apparent peptide loss from incomplete digestion of HRP protein and variability in sample handling at each site, as study complexity was increased. Study I represents the optimum assay performance, as synthetic peptides (not proteins) were used as analytes. Protein digestion in study II (at a central location in the absence of plasma) and study III (at individual sites and in the presence of plasma) introduces potential sources of sample loss that decrease analyte recovery and reduce measured concentrations for studies II and III. Intralaboratory CVs for studies I and II constitute a measure of the technical variation due to instrument and data acquisition, as all sample preparation was performed centrally. The intralaboratory CVs at each analyte concentration point are shown in Figure 5b for the HRP-SSD peptide with color-coded markers representing individual laboratories. Similar calculations and plots are derived for the other 9 peptides.

The results are summarized in Table 5. Intralaboratory precision is represented by the median CV calculated from all concentration points for a particular peptide (based on quadruplicate measurements for a single chosen transition) for each site, and for each study. The interlaboratory precision is represented by the median CV calculated at each concentration point for a particular peptide across all sites and for each study. In Table 5 the interlaboratory precision at a concentration close the LOQ is shown. The CV calculations at each concentration point for a peptide at a given laboratory is based on four replicates for studies I and II and on 12 data points (four technical replicates for each of the three process replicates) for study III.

**Table 5:** Summary of Results for Studies I, II, and III (combined results for process replicates a, b, c) for each peptide across sites for inter-site CV, intra-site CV, linear slope and % recovery.

Signature Peptide	Study I				Study II				Study III^a^			
	
	Inter- site CV	Intra- site CV	Linear Slope	% Recov.	Inter- site CV	Intra- site CV	Linear Slope	% Recov.	Inter- site CV	Intra- site CV	Linear Slope	% Recov.
APR-AGL	9.20%	3.9-11.2%	1.157	114.5	13.10%	2.0-7.8%	0.575	57.5	13.70%	7.3-45.2%	0.738	79.4
CRP-ESD	5.90%	2.2-5.9%	1.124	118.4	10.50%	3.1-8.4%	0.573	61.4	16.70%	8.5-18.1%	0.439	48.9
CRP-GYS	5.40%	1.4-10.2%	1.324	140.5	5.60%	1.2-6.4%	0.546	56	18.50%	6.6-35.0%	0.159	18.5
HRP-SSD	14.10%	4.0-8.9%	1.198	120.4	5.50%	4.6-7.3%	0.794	82.3	21.90%	8.4-21.4%	0.43	45.7
LEP-IND	12.50%	2.9-10.3%	1.163	119.1	29.50%	2.6-15.3%	0.152	14.9	50.40%	11.7-54.9%	0.242	25.6
MBP-HGF	4.30%	1.7-6.3%	1.161	118.6	9.30%	1.5-7.8%	0.758	77.3	21.80%	7.4-32.8%	0.238	23.8
MBP-YLA	5.10%	2.1-9.3%	1.275	130.3	4.10%	1.5-14.1%	0.806	83.8	N/A	N/A%	N/A	> 1.0
MYO-LFT	4.90%	1.6-5.7%	1.518	154.4	3.80%	2.0-6.3%	1.012	101.3	23.10%	8.9-21.6%	0.504	60.4
PSA-IVG	6.90%	1.3-14.7%	1.658	165.4	5.50%	2.0-11.2%	0.848	81.9	17.20%	7.6-13.7%	0.587	58
PSA-LSE	8.90%	1.2-6.9%	1.098	111.4	5.30%	2.0-4.6%	1.524	151.3	10.30%	7.6-13.7%	0.918	92.7

The CVs increase in distribution between studies I, II, and III as expected with increasing complexity of the three studies. Adapted from Addona, et. al., Nature Biotechnology, 27(7):633-41 (25).

In this analysis, the interlaboratory precision is calculated as the median intralaboratory CV. While this measure summarizes the precision obtained across multiple laboratories, it does not account for the accuracy of the measurement across different laboratories--all the laboratories may have repeated measurements that are very close (high precision, and hence low CV), but the actual measurements may differ significantly from laboratory to laboratory (poor accuracy). Hence, in clinical domains, the interlaboratory precision is calculated as the CV of all the measurements of a peptide (at a concentration) across all the laboratories [20]. An additional study investigated the use of more sophisticated mixed effect models to evaluate the sources of variation in the NBT study [42].

## Discussion

For researchers new to SID-MRM-MS assays, this section outlines important aspects of the experimental design and data analysis, along with practical tips.

When constructing a calibration curve, attempt to use a concentration range that extends past the estimated LOD and upper LOQ so that these Figures of Merit can be calculated from the data. Prepare the calibration curve in a matrix that is identical to that of the actual sample in order to accurately reproduce the chemical noise contributions from the matrix. If this is not possible, use a matrix that is very similar in composition. Analyze matrix blank samples periodically throughout the assay. This will provide the best determination of the signal-to-noise of the sample matrix and internal standards, and detect any potential for analyte carryover that would be encountered in a quantitative assay of unknown samples.

To determine the technical variability of an assay, analyzing a minimum of 3 technical replicates (repeat injections from the same sample) is suitable. The use of process replicates (preparations of the samples made at different times) can be used to calculate the analytical variability of an assay. Usually, technical variability is smaller than analytical variability. A minimum of 3 replicates should be prepared for each concentration point in calibration curves. The precision of the calculations improves with increased sample size, so if time and resources permit, more replicates are favorable.

Most methods of calculating the LOD or LOQ use the calibration curve data points to interpolate the determined value. To make sure the calculated LOD seems reasonable, it is recommended to visually inspect the individual concentration points to make sure the calculated values make sense and the concentration point above the calculated LOD is easily discernable. The main factors affecting the calculated LOD of an assay are the noise present in the matrix blank, and the reproducibility of that noise. Matrices that have a lot of noise and/or where the measurement of that noise is very variable will result in higher LODs.

Often in practice, the largest influence on the sensitivity of an assay is not the instrument itself, but how well the instrument is performing. Variability can have a profound impact on sensitivity. Evaluating the reproducibility of an LC-MRM-MS system is highly recommended before evaluating its sensitivity. This can be accomplished by making repeat measurements of the same sample using the same method, to achieve CV values less than 20%.

Last but not least, automated data processing tools and algorithms should be applied with care, continually assessing data quality, consistently accounting for outliers, and monitoring results.

## Conclusion

MRM-MS assays are increasingly being deployed to measure and quantify peptides (and hence, proteins) in a variety of matrices and backgrounds. This manuscript provides a complete toolkit for the analysis and interpretation of MRM-MS experiments.

Sound statistical analysis of MRM-MS data starts with high quality data. Using algorithms like AuDIT and mProphet (Section 5), the data quality assessment can be automated resulting in a more reliable high throughput analysis pipeline which quickly weeds out poor quality transitions or transitions with interferences.

Calibration and characterization of detection limits and variability are important aspects of any quantitative assay. We present a comparative set of methods and approaches for MRM-MS assay calibration, regression analysis, determination of confidence intervals, dealing with endogenous signal, assessment of detection limits and multi-laboratory characterization of assay performance and precision.

While systematic and principled analysis of data is essential for achieving the full potential of quantitative MRM-MS assays, care has to be exercised in experiment design and data generation to maximize reproducibility and data quality. There are many experimental and other variables beyond the scope of this manuscript that need to be addressed for successful deployment and use. Several new multi-laboratory studies aim to circumscribe and control these aspects. Two such factors worth mentioning are (i) digestion and (ii) system suitability assessment. Reproducible digestion of proteins is a pre-requisite for reliable quantification using MRM-MS. Several on-going studies attempt to not only determine standard operating procedure to ensure proper digestion, but also use specially chosen marker peptides to detect improper or incomplete digestion. Furthermore, given the complexity of chromatography and MS instrumentation, constant assessment of optimal system performance is necessary to guarantee data quality[43]. Studies for defining, assessing and maintaining system suitability are also under way. Most of these large multi-laboratory studies are being carried out under the auspices of the Clinical Proteomics Technology Assessment for Cancer (CPTAC) program sponsored by the National Cancer Institute (http://proteomics.cancer.gov), with the overarching goal of advancing biomarker discovery and enabling the advancement of promising new technologies like MRM-MS towards clinically deployed assays.

## Competing interests

The authors declare that they have no competing interests.
